# Targeting of miR-93-5p/Mfn2 Axis Attenuates Lung Fibrosis in Rats With Acute Respiratory Distress Syndrome by Regulating Endoplasmic Reticulum Stress

**DOI:** 10.33549/physiolres.935623

**Published:** 2025-10-01

**Authors:** Xiaoxia ZHANG, Tao LIU, Zhong DAI, Qin ZHAO, Ning AN

**Affiliations:** 1 Department of Critical Care Medicine, Wuhan Fourth Hospital, Wuhan, Hubei Province, China; 2 Graduate School, Hubei University of Medicine, Shiyan, Hubei Province, China; 3 Institute of Anesthesiology and Critical Care, Union Hospital, Tongji Medical College, Huazhong University of Science and Technology, Wuhan, Hubei Province, China

**Keywords:** Acute respiratory distress syndrome, miR-93-5p, Pulmonary fibrosis, Endoplasmic reticulum stress, Mitofusin 2

## Abstract

High mortality rates among patients with acute respiratory distress syndrome (ARDS) have been linked to pulmonary fibrosis. MicroRNAs exhibit significant potential in modulating pulmonary fibrosis. However, the specific role and underlying mechanisms of miR-93-5p in the context of ARDS-associated pulmonary fibrosis remain largely unexplored. Mitofusin 2 (Mfn2) is a highly conserved transmembrane GTPase. Our previous study demonstrated that the upregulation of Mfn2 can inhibit pulmonary fibrosis in ARDS mice. In this investigation, we identified upstream miRNAs regulating Mfn2 using bioinformatics tools such as TargetScan, miRDB, and microT-CDS. Based on the expression levels of these miRNAs in lung tissue from rats with LPS-induced ARDS, miR-93-5p was selected as the focus of our research. We modulated miR-93-5p expression in ARDS rats *via* tail vein injection of a miR-93-5p antagomir. Thereafter, we conducted pathological staining and molecular assays to examine the impact of miR-93-5p on pulmonary fibrosis in ARDS rats and to elucidate its potential mechanisms. The results demonstrated that the expression of miR-93-5p was significantly upregulated in the lung tissue of ARDS rats. LPS-induced ARDS rats exhibited severe pulmonary fibrosis, inflammation, and strong endoplasmic reticulum (ER) stress. Furthermore, Mfn2 expression exhibited a negative correlation with miR-93-5p expression. Inhibition of miR-93-5p markedly upregulated Mfn2 expression, attenuated ER stress and lung inflammation, and decreased collagen deposition. In conclusion, the inhibition of miR-93-5p upregulated Mfn2 expression and attenuated ER stress, consequently ameliorating pulmonary fibrosis in ARDS rats.

## Introduction

Acute respiratory distress syndrome (ARDS) is a clinical critical illness characterized by progressive respiratory distress and refractory hypoxemia. The mortality rate among patients with ARDS is as high as 46.1 % [1], and those who do survive often face a diminished quality of life attributable to compromised lung function [2]. Therefore, ARDS poses a significant threat to public health and human life. However, there remains a notable absence of a clinical gold standard for the diagnosis of ARDS. In addition, owing to the heterogeneity in the etiology of ARDS, there remains a deficiency in clearly defined and effective pharmacological treatments in clinical practice [3].

The morphological evolution of lung injury in ARDS can be categorized into three phases: the exudative phase, the proliferative phase, and the fibrotic phase [4]. Researches have demonstrated that pulmonary fibrosis is not confined to the late stages of ARDS but can also manifest in the early stages [5,6]. Furthermore, pulmonary fibrosis is a critical factor contributing to the high mortality rate among ARDS patients following the acute phase [7]. Thus, mitigating ARDS-related pulmonary fibrosis is a critical strategy to enhance the prognosis of patients with ARDS.

Endoplasmic reticulum (ER) is a cellular organelle primarily responsible for maintaining protein homeostasis. When the normal processing of proteins within the ER is disrupted, leading to the accumulation of misfolded proteins, this condition is referred to as ER stress [8]. ER stress occurs in alveolar epithelial cells, which causes dysfunction of epithelial cells and affects epithelial-mesenchymal transition and myofibroblast differentiation, thereby promoting fibrotic reconstruction [8,9]. Mitofusin 2 (Mfn2) is a highly conserved transmembrane GTPase that resides in both the outer mitochondrial membrane and the endoplasmic reticulum. Beyond its role as a key regulator of mitochondrial fusion, Mfn2 also facilitates the interaction between mitochondria and the endoplasmic reticulum [10]. It has been demonstrated that the activation of the Mfn2 pathway can mitigate ER stress [11–13]. In our previous research, we demonstrated that Mfn2 is expressed in human lung fibroblasts and that its overexpression inhibits the proliferation of these cells [14]. Furthermore, we observed that upregulation of Mfn2 expression promotes mitophagy in the lung tissues of mice with ARDS-associated pulmonary fibrosis, thereby impeding the progression of pulmonary fibrosis [15]. These studies indicate that Mfn2 represents a significant and promising therapeutic target for mitigating ARDS-associated pulmonary fibrosis. However, it remains to be determined whether the mechanism by which Mfn2 influences lung fibrosis progression in ARDS is mediated through the regulation of ER stress.

miR-93 originates from the miR-106b-25 gene cluster located on chromosome 7q22. The overexpression of miR-93-5p significantly enhances tumor cell proliferation and inhibits apoptosis, thereby contributing substantially to carcinogenesis and tumor progression [16,17]. However, the specific role of miR-93-5p in the pathological process of pulmonary fibrosis in ARDS remains unclear. Notably, miR-93 has been demonstrated to facilitate the proliferation and migration of vascular smooth muscle cells through the regulation of Mfn2 [18]. Furthermore, utilizing the predictive analysis capabilities of the online software TargetScan, we identified a miR-93-5p binding site within the 3′ untranslated regions (UTR) of Mfn2. Therefore, we hypothesized that miR-93-5p may contribute to the development of ARDS-related pulmonary fibrosis by targeting Mfn2.

In this study, we examined the role of miR-93-5p in pulmonary fibrosis in a rat model of ARDS and elucidated its underlying mechanisms *in vivo*. The objective of this study is to elucidate the molecular mechanisms underlying pulmonary fibrosis in ARDS, thereby providing a theoretical foundation for the development of effective therapeutic strategies for this condition.

## Materials and Methods

### Bioinformatics analysis

Potential rat-derived miRNAs targeting Mfn2 were analyzed using the online websites TargetScan (https://www.targetscan.org/vert_72/), miRDB (https://mirdb.org/), and microT-CDS (https://diana.e-ce.uth.gr/). The miRNAs acquired from the aforementioned three databases were subjected to cross-analysis, resulting in a Venn diagram. Subsequently, miRNAs at the intersection were screened to obtain the research target, miR-93-5p.

### Rat model of ARDS

Specific pathogen free (SPF) male adult Sprague-Dawley (SD) rats (body weight 200~220 g) were supplied by the SPF (Beijing) Biotechnology Co., Ltd. Following one week of adaptive feeding, the ARDS model was induced using lipopolysaccharide (LPS, from Escherichia coli O111:B4; Sigma, St. Louis, USA). The detailed procedures were as follows [19]: The modeling process commenced at 9:00 AM, and the rats were anesthetized *via* intraperitoneal injection of 2 % sodium pentobarbital (45 mg/kg). Subsequently, the trachea was exposed, and LPS (5 mg/kg) was slowly instilled. After closing the incision, the rats were maintained in an upright position and gently rotated to ensure uniform distribution of LPS within the lungs. 8 h post-modeling, the rats exhibited signs including tachypnea, lethargy, and reduced mobility, which confirmed the successful establishment of the model. The rats in the control group were administered the same volume of normal saline *via* tracheal instillation as the model rat, and all other procedures were conducted in an identical manner. Following 24 h of modeling, lung tissue samples from six model rats and six control rats were collected to detect the expression levels of five potential miRNAs targeting Mfn2.

**Fig. 1:**
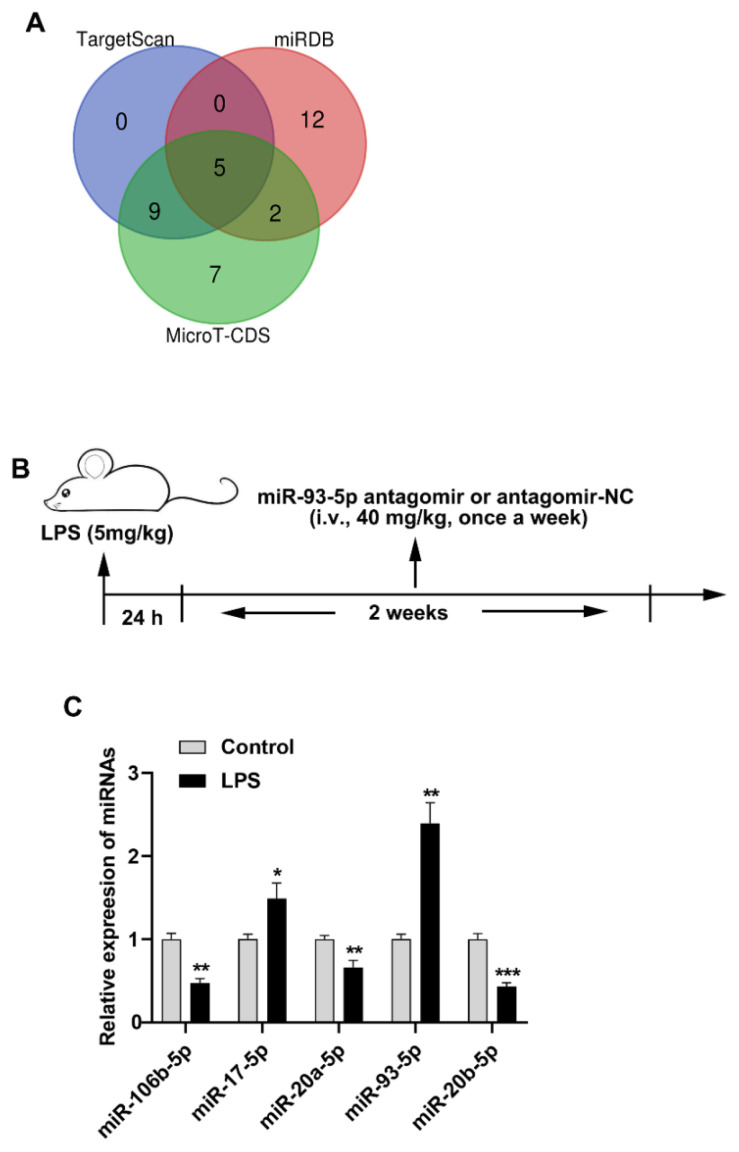
miR-93-5p is highly expressed in the lung tissue of LPS-induced ARDS rats. (**A**) The Venn diagram illustrates the miRNAs targeting Mfn2 as obtained from the TargetScan, miRDB, and microT-CDS databases. (**B**) Timeline of animal experiments. (**C**) The expression levels of rno-miR-106b-5p, rno-miR-17-5p, rno-miR-20a-5p, rno-miR-93-5p and rno-miR-20b-5p in the lung tissue of rats were detected using qRT-PCR (n=6). * *p*<0.05, ** *p*<0.01, *** *p*<0.001.

After 24 h of modeling, the model rats were randomly allocated into three groups: the LPS group, the LPS+antagomir-NC group, and the LPS+miR-93-5p antagomir group. miR-93-5p antagomir or antagomir-NC (40 mg/kg [20]; RiboBio, Guangzhou, China) was injected into the tail vein of rats once a week for 2 consecutive weeks. The equal volume of normal saline was injected *via* the tail vein in the control and LPS groups. The timeline of the animal experiments is presented in Figure 1B. The mortality rate among rats during the modeling and administration phases was approximately 20 % (no mortality was observed in the control group). To ensure consistency, deceased rats were excluded from the study, allowing for a final cohort of 12 rats per group to be used for subsequent detection and analysis. Upon completion of the grouping treatment, the rats were euthanized *via* anesthetic overdose. Bronchoalveolar lavage fluid (BALF) was collected from 6 rats in each group, while lung tissues were obtained from the remaining 6 rats. One-half of the lung tissues were fixed in 4 % paraformaldehyde for histological analysis, and the other half were stored at −80 °C for subsequent biochemical or molecular assays. The rat trachea was exposed, and 5 ml of precooled PBS was drawn into a syringe and then slowly injected into the lungs through the tracheal cannula. After allowing the fluid to remain in the lungs for 30 s, the syringe was gently withdrawn to collect the lavage fluid. This lavage procedure was repeated three times, and the collected lavage fluids were combined and centrifuged to obtain the supernatant. All animal experiments conducted in this study adhered strictly to the Laboratory Animal-Guideline for ethical review of animal welfare issued by the Ministry of Science and Technology of China, and obtained ethical approval from the Laboratory Animal Welfare and Ethics Committee of Bestcell Model Biological Center (Issue No. BSMS-2024-12-03C).

### Histopathological staining

The fixed rat lung tissues were removed and embedded in paraffin wax to complete the sections. Thereafter, the lung tissue sections underwent deparaffinization followed by a series of pathological staining procedures.

For hematoxylin-eosin (HE) staining, lung tissue sections were sequentially stained with hematoxylin and eosin according to the kit instruction provided by the manufacturer (Servicebio, Wuhan, China). The sections were then sealed with neutral gum and examined under a microscope to observe the pathological changes in the lung tissues. Each parameter was independently scored on a scale ranging from 0 (absent) to 4 (severe), based on assessments of alveolar wall thickness, inflammatory cell infiltration, and pulmonary hemorrhage within the visual field, with the sum of these scores representing the overall lung injury score [21].

For Masson staining, lung tissue sections were stained according to the reagent instruction provided by the manufacturer (Servicebio, Wuhan, China). Specifically, the tissue sections were first immersed in 2.5 % potassium dichromate solution overnight. Subsequently, the sections were sequentially stained with Hematoxylin, Ponceau acid fuchsin, 1 % phosphomolybdic acid, and 2.5 % aniline blue. Following differentiation by rinsing with 1 % acetic acid, the sections were dehydrated using absolute ethanol and then sealed with neutral gum. Finally, the staining of lung tissue sections was examined under a microscope. The collagen fibers appeared sky blue or dark blue, whereas muscle fibers and cellulose stained red or purplish red. The area of the blue-stained region (collagen) and the total tissue area within the field of view were analyzed using Image Pro Plus software, and the collagen volume fraction (CVF) was subsequently calculated.

For terminal deoxynucleotidyl transferase dUTP nick end labeling (TUNEL) staining, tissue sections were treated with proteinase K for repair, followed by the addition of membrane permeabilization solution and incubation at room temperature for 20 min. Following the addition of the TUNEL reagent (Servicebio, Wuhan, China) and incubation at 37 °C for 1 h, the nuclei were counterstained with DAPI (Servicebio, Wuhan, China). After sealing, the sections were examined under a fluorescence microscope to observe cell apoptosis in lung tissues. The Image Pro Plus software was utilized to quantify the number of apoptotic cells exhibiting green fluorescence and the number of nuclei displaying blue fluorescence within the field of view, followed by the calculation of the proportion of apoptotic cells.

### Immunohistochemical analysis

The lung tissue sections of rats were dewaxed, and then endogenous peroxidase activity was blocked with 3 % hydrogen peroxide. 3 % bovine serum albumin (BSA; Sigma, Sigma, St. Louis, USA) was dropped on the tissue and incubated at room temperature for 30 min. The anti-Mfn2 antibody (1:100; Abcam, Cambridge, UK) was added and incubated at 4 °C overnight. Then, horseradish peroxidase (HRP)-labeled secondary antibody (1:1000; Abcam, Cambridge, UK) was added and incubated at room temperature for 50 min. DAB chromogenic solution (Servicebio, Wuhan, China) was added, and when positive staining was observed as brownish yellow under a microscope, the staining was terminated by rinsing with running water. After counterstaining with hematoxylin solution, dehydration and sealing were performed. Ultimately, the positive expression of Mfn2 in lung tissue within the field of view was observed microscopically (tan-stained regions), and the integrated optical density (IOD) of these regions was analyzed using Image Pro Plus software.

### Enzyme-linked immunosorbent assay (ELISA)

The collected rat BALF was centrifuged at 1500 RPM for 10 min to obtain the supernatant. The concentrations of interleukin-1 beta (IL-1β), interleukin-6 (IL-6), and tumor necrosis factor-alpha (TNF-α) in the BALF were quantified according to the rat ELISA kits instructions provided by the manufacturer (Elabscience, Wuhan, China).

### Biochemical assay

Rat lung tissue was collected in a test tube, and 1 ml of hydrolysate was added. The mixture was then hydrolyzed at 95 °C for 20 min. Following this, the pH of the sample solution was adjusted to neutrality. Subsequently, the hydroxyproline (Hyp) content in the lung tissue was determined according to the manufacturer’s instructions provided with the Hyp assay kit (Nanjing Jiancheng Bioengineering Institute, Nanjing, China).

### Quantitative real-time polymerase chain reaction (qRT-PCR)

Total RNA was extracted from rat lung tissue utilizing RNAiso reagent (TaKaRa, Dalin China). Subsequently, RNA was reverse transcribed to synthesize cDNA using the microRNA Reverse Transcription Kit and the RevertAid First Strand cDNA Synthesis Kit (Thermo Fisher Scientific, Waltham, USA), respectively. Finally, qRT-PCR was performed using the Power Track SYBR Green Master Mix (Thermo Fisher Scientific, Waltham, USA) with cDNA as the template. The reaction protocol was as follows: initial denaturation at 95 °C for 2 min, followed by 40 cycles consisting of denaturation at 95 °C for 15 s, annealing at 58 °C for 20 s, and extension at 72 °C for 30 s. The specific primers sequences utilized in this experiment were presented in Table 1. The relative expression levels of miR-93-5p and Mfn2 mRNA in lung tissues were determined using the 2^−ΔΔCt^ method, with U6 and GAPDH serving as internal references, respectively.

### Western blotting

Rat lung tissue was dissected into small fragments, followed by the addition of lysis buffer to extract total protein. The protein concentration was quantified using a BCA assay and subsequently subjected to electrophoresis for separation. The target proteins were transferred from the gel to polyvinylidene fluoride (PVDF) membranes (Millipore, Billerica, USA) and blocked with 5 % skim milk at room temperature for 2 h. The membranes were then incubated with primary antibodies overnight at 4 °C. Following this, the membranes were incubated with HRP-labeled secondary antibodies (1:2000; Abcam, Cambridge, UK) for 1 h at room temperature. Finally, chemiluminescence reagents were applied dropwise onto the membranes for visualization. The primary antibodies utilized in this experiment were purchased from Abcam (Cambridge, UK) as follows: anti-Mfn2 antibody (1:2000), anti-transforming growth factor beta l (TGF-β1) antibody (1:1000), anti-alpha-smooth muscle actin (α-SMA) antibody (1:1000), anti-Collagen I antibody (1:1000), anti-activating transcription factor 4 (ATF4) antibody (1:500), anti-CCAAT/enhancer-binding protein homologous protein (CHOP) antibody (1:1000), anti-protein kinase R-like ER kinase (PERK) antibody (1:1000), anti-phosphorylated PERK (p-PERK) antibody (1:1000), and anti-GAPDH antibody (1:10000).

### Dual-luciferase reporter assay

Based on the predicted binding site between miR-93-5p and Mfn2 3’UTR on the TargetScan website, we constructed dual luciferase reporter gene vectors containing the wild-type or mutant sequences of the Mfn2 3’UTR, designated as Mfn2-wt and Mfn2-mut, respectively. Rat glioma C6 cells (National Collection of Authenticated Cell Cultures, Shanghai, China) in the logarithmic growth phase were seeded into 6-well cell culture plates at a density of 3×10^5^ cells per well. Transfection experiments were conducted once the cell confluence reached approximately 70 %. Mfn2-wt or Mfn2-mut, along with miR-93-5p mimics (sense: 5′-CAAAGUGCUGUUCGUGCAGGUAG-3′ and anti-sense: 5′-CUACCUGCACGAACAGCACUUUG-3′) or negative control mimics (mimics-NC, sense: 5′-CAAAGUGCUGUUCGUGCAGGUAC-3′ and anti-sense: 5′-GUACCUGCACGAACAGCACUUUG-3′), were co-transfected into C6 cells using Lipofectamine 2000 reagent (Invitrogen, Carlsbad, USA). 24 h post-transfection, alterations in cellular luciferase activity were detected according to the instruction of the dual-luciferase reporter gene assay kit provided by the manufacturer (Beyotime, Shanghai, China).

### Statistical analysis

In this study, statistical analysis of the experimental results was conducted using SPSS 23.0 and GraphPad Prism 8 software, with the experimental data presented as mean ± standard deviation (SD). An independent samples *t*-test was employed to compare the two groups. For comparisons among multiple groups, one-way analysis of variance (ANOVA) followed by Tukey’s *post hoc* test was utilized. A p-value of less than 0.05 was deemed statistically significant.

## Results

### miR-93-5p is highly expressed in the lung tissue of LPS-induced ARDS rats

Our previous studies has demonstrated that Mfn2 is downregulated in the lung tissues of ARDS mice, and overexpression of Mfn2 can alleviate pulmonary fibrosis, suppress the proliferation of lung fibroblasts, and decrease collagen production in ARDS mice [15]. To further explore the molecular mechanism by which Mfn2 participates in regulating the formation of pulmonary fibrosis in ARDS, we utilized online prediction tools to analyze potential miRNAs targeting Mfn2 upstream. Through this analysis, we obtained 14, 19 and 23 miRNAs targeting Mfn2 from the TargetScan, miRDB and microT-CDS respectively. After performing the intersection analysis, 5 miRNAs were obtained, namely rno-miR-106b-5p, rno-miR-17-5p, rno-miR-20a-5p, rno-miR-93-5p and rno-miR-20b-5p (Fig. 1A). LPS-induced rats were utilized to establish an *in vivo* model of ARDS (Fig. 1B). The results demonstrated that the expression levels of miR-17-5p and miR-93-5p were significantly upregulated in lung tissues of ARDS0 rats, whereas those of miR-106b-5p, miR-20a-5p, and miR-20b-5p were markedly downregulated (Fig. 1C). In our prior investigation, we examined the biological role of miR-17 in ARDS mice [15]. Consequently, miR-93-5p was selected as the focus of this study.

### Inhibition of miR-93-5p attenuates lung injury and inflammation in LPS-induced ARDS rats

**Fig. 2:**
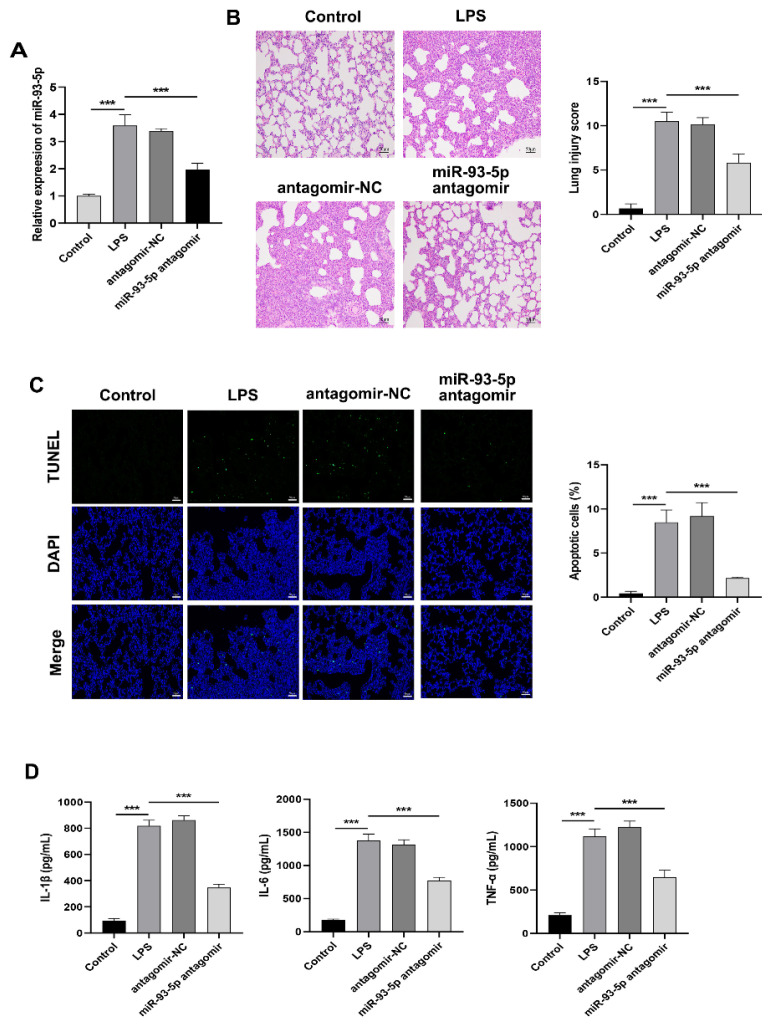
Effect of inhibiting miR-93-5p on lung injury in LPS-induced ARDS rats. (**A**) The expression of miR-93-5p in the lung tissue of rats was detected using qRT-PCR. (**B**) The pathological alteration in lung tissue of rats was observed using HE staining. Scale bar=50 μm. (**C**) The level of apoptosis in lung tissue of rats was assessed using TUNEL staining. Scale bar=50 μm. (**D**) The concentrations of IL-1β, IL-6 and TNF-α in BALF of rats were quantified using ELISA. n=6. *** p<0.001.

In order to investigate the role of miR-93-5p in pulmonary fibrosis development in ARDS, we silenced the expression of miR-93-5p in an LPS-induced ARDS rats (Fig. 2A). Subsequently, we conducted a histopath-ological observation of the rat lungs. The results showed that the lung tissue structure in the control group was intact, with no significant pathological features and minimal apoptosis. In contrast, LPS administration resulted in severe pulmonary injury characterized by thickened alveolar walls, edema, hemorrhage, rupture, inflammatory cell infiltration, and increased apoptosis. Notably, inhibition of miR-93-5p mitigated the LPS-induced pathological damage and reduced apoptotic cells in the lung tissue (Fig. 2B–C). Furthermore, we also found that the levels of inflammatory cytokines IL-1β, IL-6 and TNF-α were significantly increased in BALF of LPS-induced ARDS rats, while miR-93-5p silencing reduced the levels of these pro-inflammatory cytokines (Fig. 2D). These findings suggest that the inhibition of miR-93-5p attenuates lung injury and inflammatory response in rats with LPS-induced ARDS.

### Inhibition of miR-93-5p ameliorate lung fibrosis after lung injury in LPS-induced ARDS rats

**Fig. 3:**
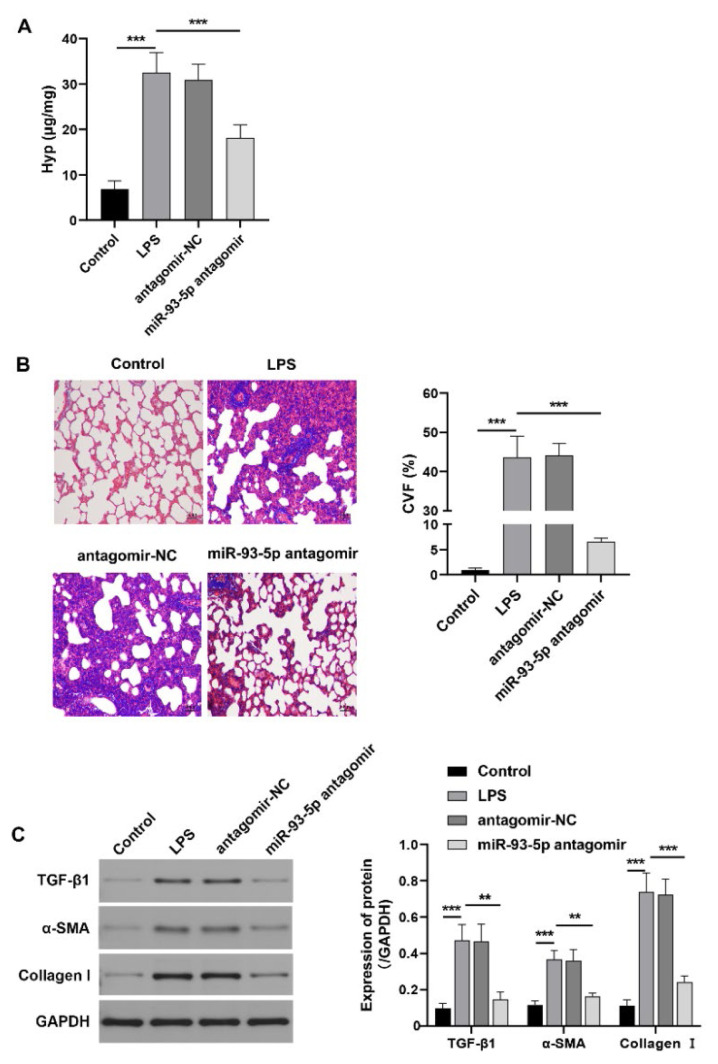
Effect of inhibiting miR-93-5p on pulmonary fibrosis in LPS-induced ARDS rats. (**A**) The content of Hyp in lung tissue of rats. (**B**) The distribution of collagen fibers in lung tissue of rats was assessed using Masson staining. Scale bar=50 μm. (**C**) The protein expression levels of TGF-β1, α-SMA, and collagen I in lung tissue of rats were detected using Western blotting. n=6. ** p<0.01, *** p<0.01.

Next, we conducted experimental analyses of lung fibrosis-related indices in rats. The results demonstrated that the Hyp content in lung tissue from LPS-induced rats was significantly elevated (Fig. 3A). Additionally, blue-stained collagen fibers were observed in lung tissues, and the expression levels of TGF-β1, α-SMA, and collagen I were markedly increased (Fig. 3B–C). These data suggest that rats with LPS-induced ARDS exhibit significant pulmonary fibrosis. In contrast, inhibition of miR-93-5p significantly decreased the content of Hyp and reduced the protein expression levels of TGF-β1, α-SMA, and Collagen I in the lung tissue of ARDS rats. In addition, the distribution of collagen fibers in the lung tissue was diminished. These findings suggest that down-regulation of miR-93-5p can mitigate pulmonary fibrosis in LPS-induced ARDS rats.

### Inhibition of miR-93-5p blocks the activation of ER stress in LPS-induced ARDS rats

**Fig. 4:**
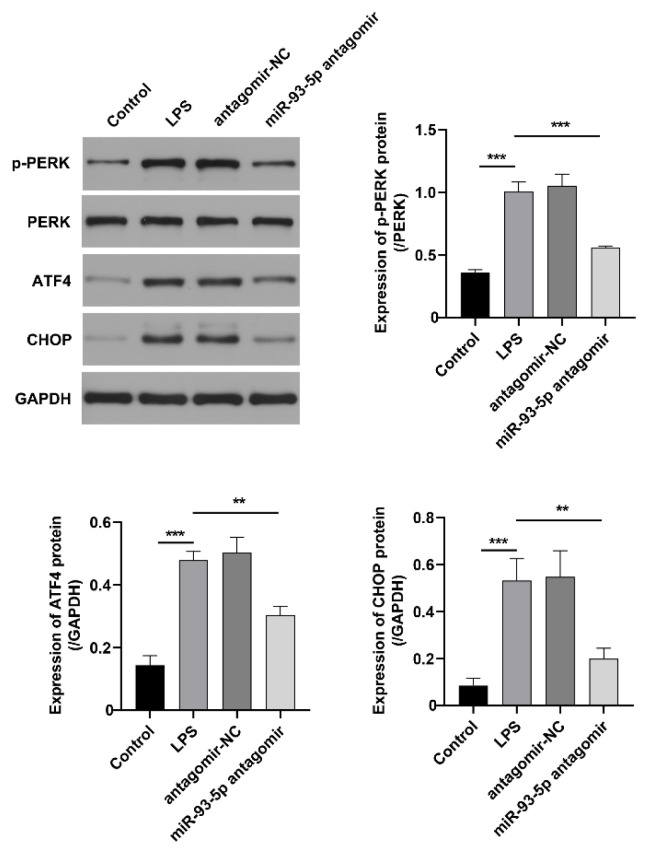
Effect of inhibiting miR-93-5p on ER stress in LPS-induced ARDS rats. The protein expression levels of PERK, p-PERK, ATF4, and CHOP in lung tissue of rats. n=6. ** p<0.01, *** p<0.001.

To investigate the impact of miR-93-5p on ER stress in lung tissues of rats with LPS-induced ARDS, we measured the expression levels of associated proteins. The results demonstrated that the phosphorylation level of PERK, as well as the protein expression levels of ATF4 and CHOP, were markedly elevated in the lung tissues of LPS-induced ARDS rats. However, inhibition of miR-93-5p significantly attenuated the expression levels of these ER stress-associated proteins in the lung tissues of LPS-induced rats (Fig. 4). These findings indicate that the down-regulation of miR-93-5p attenuates ER stress in the lung tissue of LPS-induced ARDS rats.

### miR-93-5p negatively regulates Mfn2 expression in LPS-induced ARDS rats

**Fig. 5:**
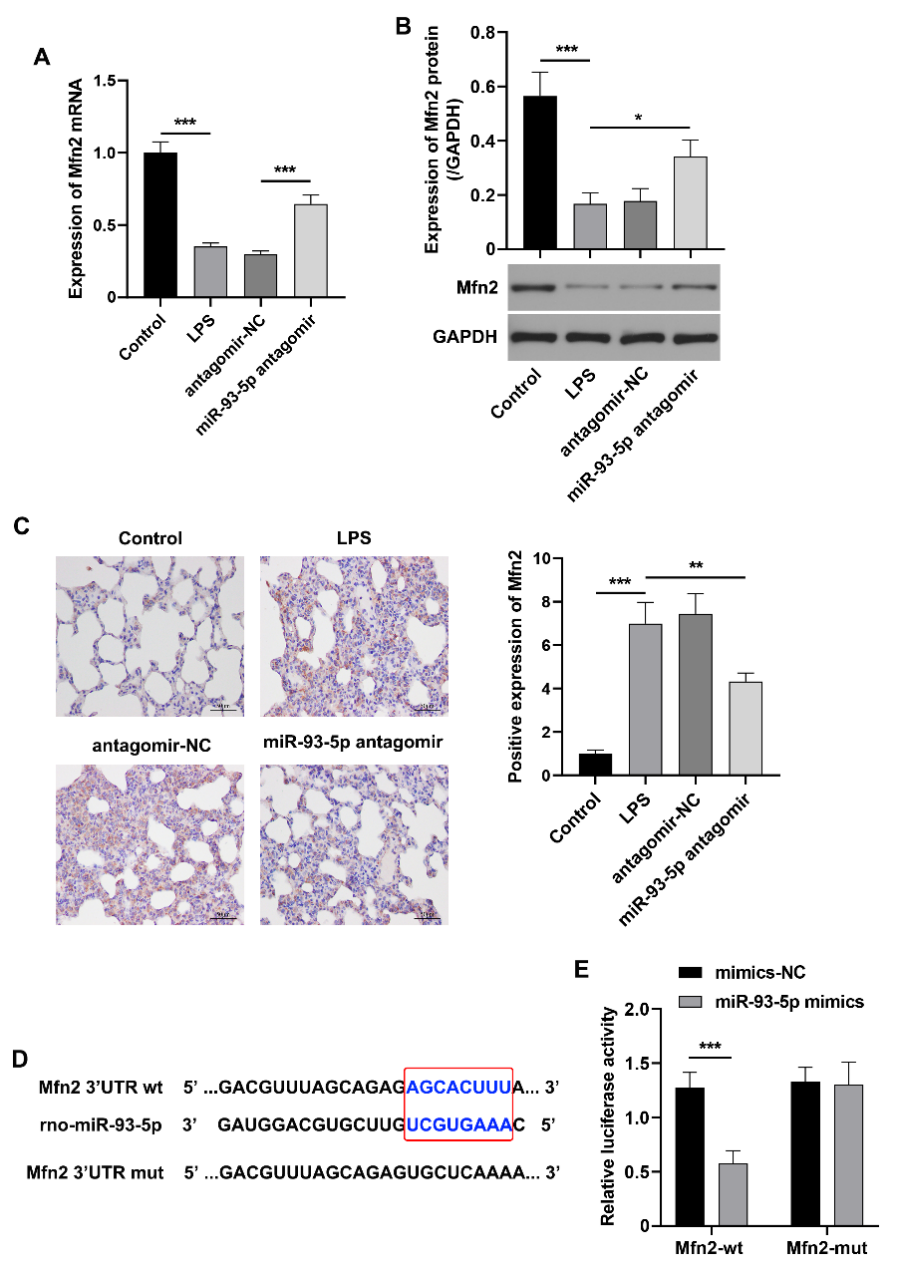
miR-93-5p targets Mfn2 in the lung tissue of LPS-induced ARDS rats. (**A**) The mRNA expression level of *Mfn2* in the lung tissue of rats was detected using qRT-PCR (n=6). (**B**) The protein expression level of Mfn2 in the lung tissue of rats was detected using Western blotting (n=6). (**C**) The expression of Mfn2 in the lung tissue of rats was observed using immunohistochemical staining (n=6). Scale bar=50 μm. (**D**) The binding site between miR-93-5p and the 3′UTR of Mfn2. (**E**) The targeted regulatory relationship between miR-93-5p and Mfn2 was verified using dual-luciferase reporter assay (n=3). * p<0.05, ** p<0.01, *** p<0.001.

Consistent with the previous findings [15], the results of the present study also showed that the expression levels of Mfn2 mRNA and protein were significantly reduced in the lung tissues of rats with LPS-induced ARDS. Conversely, Mfn2 expression was upregulated following the down-regulation of miR-93-5p expression in the lung tissues of LPS-induced rats (Fig. 5A–C). Furthermore, based on the predicted binding sites between miR-93-5p and the 3′-UTR of Mfn2 obtained from the TargetScan database (Fig. 5D), a dual-luciferase reporter assay was conducted. The result confirmed that Mfn2 is a downstream target gene of miR-93-5p (Fig. 5E). It is proposed that miR-93-5p may contribute to lung injury and pulmonary fibrosis in ARDS rats by down-regulating Mfn2 through direct targeting.

## Discussion

Despite the high global morbidity and mortality associated with ARDS, there is still a lack of specific drugs to repair lung damage. Currently, the therapeutic strategy for ARDS primarily focuses on providing supportive care measures, including low tidal volume ventilation, extracorporeal membrane oxygenation, and fluid management [22]. Therefore, the development of targeted therapies for ARDS is critically important. Our previous study revealed that Mfn2 possesses significant potential to inhibit the progression of ARDS-associated pulmonary fibrosis [15,23]. In this study, we performed a comprehensive investigation into miR-93-5p, an upstream target miRNA of Mfn2. Our findings suggest that the miR-93-5p/Mfn2 axis may be an important signaling pathway involved in the pathological process of ARDS-associated pulmonary fibrosis.

ER stress restores ER homeostasis through activation of the unfolded protein response (UPR). However, excessive or prolonged ER stress may ultimately result in apoptosis [24]. UPR signaling is activated by three ER transmembrane proteins, one of which is PERK [25]. Upon phosphorylation, PERK promotes the translation of ATF4, thereby initiating the transcription of a series of genes aimed at alleviating ER stress. As ER stress intensifies, ATF4 subsequently initiates the transcription of CHOP, thus activating the pro-apoptotic program to promote cell apoptosis. There is a close relationship between ER stress and pulmonary fibrosis. In pulmonary fibrosis, ER stress can promote disease progression by promoting apoptosis, epithelial-mesenchymal transition, inflammatory response and fibroblast activation [26]. In the current study, LPS-induced ARDS rats exhibited severe pulmonary fibrosis along with endoplasmic reticulum stress in lung tissue. Therefore, we speculate that mitigating ER stress of ARDS rats would inhibit the progression of pulmonary fibrosis.

miRNAs possess significant potential in modulating ER stress. miR-205 attenuates bleomycin-induced pulmonary fibrosis in rats by inhibiting the activation of the ER stress signaling pathway through targeting GATA binding protein 3 (GATA3) [27]. miR-30c-2-3p exacerbates ER stress in ovarian cancer cells by diminishing the protein folding capacity of the ER, ultimately leading to cell apoptosis [28]. miR-93-5p plays a crucial role in maintaining protein homeostasis and modulating translation [29]. However, the precise role of miR-93-5p in ER stress within the context of pulmonary fibrosis has been seldom documented in the literature. In this study, we report for the first time that miR-93-5p is upregulated in the pulmonary fibrosis of ARDS rats. Upon inhibition of miR-93-5p expression, the expression levels of PERK pathway-related proteins were reduced, and apoptosis was significantly increased in lung tissues of ARDS rats. These findings indicate that miR-93-5p plays a crucial role in modulating ER stress in the lung tissues of ARDS rats.

TGF-β serves as a pivotal regulator in the development of pulmonary fibrosis. It induces fibrotic changes in alveolar epithelial cells through the activation of ER stress and unfolded protein response pathways, notably the PERK/CHOP pathway [30]. Yang *et al.* reported that the ER stress response protein CHOP plays a role in pulmonary fibrosis by facilitating the TGF-β1-mediated transformation of resident lung mesenchymal/stromal cells into myofibroblasts [31]. Under PM2.5 exposure, ER stress triggers apoptosis in lung cells and excessive deposition of extracellular matrix (ECM) through the activation of the TGF-β1/Smad3 signaling pathway, thereby exacerbating pulmonary fibrosis [32]. The findings of this study demonstrated that the down-regulation of miR-93-5p led to decreased expression levels of TGF-β1 and proteins associated with collagen formation in the lung tissues of ARDS rats. Consequently, the regulatory impact of miR-93-5p on ER stress may represent a critical pathway through which it contributes to pulmonary fibrosis in ARDS.

In mammalian cells, Mfn2 serves not only as a transmembrane protein embedded in the outer mitochondrial membrane to mediate fusion between adjacent mitochondria but also forms complexes that tether mitochondria to the endoplasmic reticulum, thereby modulating cellular energy metabolism and apoptosis [33]. Furthermore, Mfn2 is an upstream regulator of the PERK pathway. Silencing Mfn2 resulted in the over-activation of the three ER stress-induced UPR branches in cells [10]. Shi *et al.* demonstrated that the upregulation of UPR markers and enhanced ER stress were observed in bovine embryos following Mfn2 inhibition [34]. Of greater significance, Mfn2 also plays a crucial role in mitigating the progression of pulmonary fibrosis in ARDS [15,23]. Consequently, we hypothesized that Mfn2 may inhibit pulmonary fibrosis through the alleviation of ER stress. In this study, we identified upstream miRNAs that regulate Mfn2 using bioinformatics tools such as TargetScan, miRDB, and microT-CDS. Subsequently, *in vivo* experiments revealed a negative correlation between Mfn2 expression and miR-93-5p levels in ARDS rats with pulmonary fibrosis. Further investigation confirmed that Mfn2 is a downstream target of miR-93-5p. Upon inhibition of miR-93-5p, Mfn2 expression was significantly upregulated. Therefore, miR-93-5p may play a regulatory role in pulmonary fibrosis in ARDS rats by targeting Mfn2; however, this hypothesis requires further validation through *in vitro* cellular experiments.

## Conclusions

**Fig. 6:**
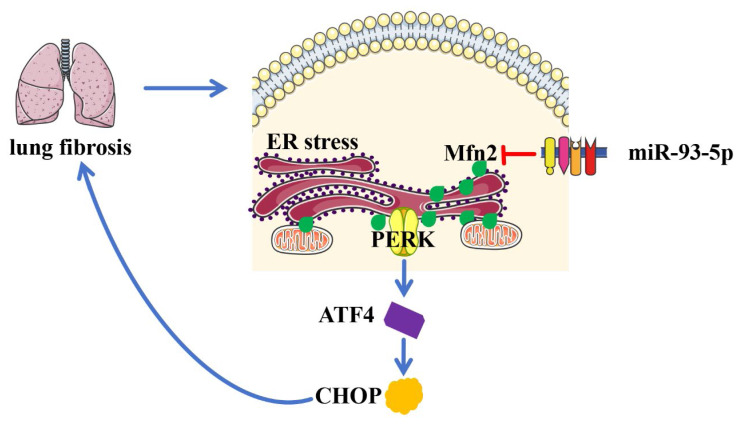
miR-93-5p participates in pulmonary fibrosis in ARDS rats by regulating ER stress through targeting Mfn2.

In conclusion, LPS-induced pulmonary fibrosis in ARDS rats was accompanied by the upregulation of miR-93-5p expression, enhanced endoplasmic reticulum stress, inflammatory response, and increased lung cell apoptosis (Fig. 6). Inhibition of miR-93-5p alleviates the aforementioned symptoms in ARDS rats, and miR-93-5p targets the regulation of Mfn2 expression. In conjunction with our previous findings, we propose that targeting the miR-93-5p/Mfn2 axis may mitigate ARDS fibrosis progression through the inhibition of ER stress.

## Figures and Tables

**Table 1:** Primer sequences used for qRT-PCR.

*Gene*	Primer	Sequence (5′-3′)
*miR-106b-5p*	Forward	TGGAGTGCTGACAGTGCAGAT
	Reverse	CTCAACTGGTGTCGTGGAGTC
*miR-17-5p*	Forward	GGCCAAAGTGCTTACAGT
	Reverse	CTCAACTGGTGTCGTGGAGTC
*miR-20a-5p*	Forward	GGGCCCTAAAGTGCTTATAGT
	Reverse	CTCAACTGGTGTCGTGGAGTC
*miR-93-5p*	Forward	GGCCAAAGTGCTGTTCGTG
	Reverse	CTCAACTGGTGTCGTGGAGTC
*miR-20b-5p*	Forward	GGCCAAAGTGCTCATAGTGC
	Reverse	CTCAACTGGTGTCGTGGAGTC
*U6*	Forward	CCTGCTTCGGCAGCACAT
	Reverse	AACGCTTCACGAATTTGCGT
*Mfn2*	Forward	TCTATGGGCATTCTCGTGGTC
	Reverse	TTCTTGCTGAACTTGGTGGCT
*GAPDH*	Forward	GCCAAGGTCATCCATGACAAC
	Reverse	GTGGATGCAGGGATGATGTTC
